# Can Sound Health Insurance Increase the Internal Circulation in the Economy of China?

**DOI:** 10.3389/fpubh.2021.710633

**Published:** 2021-07-15

**Authors:** Peng Zhao, Li-Yong Wang, Li Zhao

**Affiliations:** ^1^School of Economics and Management, Nanning Normal University, Nanning, China; ^2^Postdoctoral Fellow, Central University of Finance and Economics, Beijing, China; ^3^School of International Trade and Economics, Central University of Finance and Economics, Beijing, China; ^4^School of Continuing Education, Nanning Normal University, Nanning, China

**Keywords:** China, consumption, bootstrap rolling window causality, insurance, health

## Abstract

In 2020, President Xi Jinping put forward a constructing cycle that has been given priority in this study. This particular cycle, when considered within the inner loop and outer loop, promotes the guiding ideology of the new development pattern of the binary economy that exists in recent times. Therefore, to gauge the extent of the promotion of domestic production and consumption, from the perspectives of medical expenses, this study refers to the bootstrap rolling window causality method, which considers the evidence-based medical spending on the consumption Granger causality. The results show that the Granger causality exists between medical expenditure and consumption expenditure at different time interval endpoints. In contrast, however, the variable of consumption does not produce Granger causality between medical expenditure and consumption. In this regard, a series of measures, such as increasing medical insurance expenditure, improvement of the medical insurance system, reduction of the housing price rise, and increasing government investment have been proposed to promote the development of the domestic circular economy.

## Introduction

Since the 1990s, China has gradually integrated itself into the global value chain system by accepting industrial transfers from the United States, Japan, the Republic of Korea, and some developed countries in Europe. China has made productive and effective use of the global supply chains and marketing networks of multinational companies to develop itself into a major open economy (1, 2). The export-oriented economic model based on contract manufacturing and processing trade and the economic model dominated by foreign investment have become the main accelerating factors in the development of open economy feature in China (3, 4). In this regard, various “zones” have also been identified, which can effectively act as carriers. These include, for instance, high-tech development zones, economic development zones, export processing zones, and bonded zones, among others. However, with the rising cost of domestic production factors, the traditional development path is not as sustainable as it seems. To some extent, the export-oriented model, which is largely dominated by foreign-funded enterprises, affects the growth of domestic enterprises and hence the cultivation of the domestic market (5, 6). These factors lead to a slowdown in the development of the economy of China.

This bottleneck of the development of the open economy and the change of the external development environment requires China to gradually reduce its dependence on the global supply chain and the marketing networks of multinational corporations (7). On the other hand, it thus needs to divert itself to promote the diversification of the upstream and intermediate products supply, as well as the terminal consumption market, to build a more independent and controllable global industrial chain system. It is noteworthy that China has a complete industrial system, complete infrastructure, and a distinct development gradient among the eastern, central, and western regions that surround it. Thus, promoting and expanding domestic circulation will help strengthen their domestic supporting capacity as well. This is especially true in the context of the current outbreak of the new champions league, which has severely impacted the background of the global industrial chain and supply chain, which relies heavily on domestic circulation. This thus enhances the level of the industrial and the supply chain, which depends on the mass-market potential. It is critical to promote the high quality of the open economy development of China; as a result, the key lies in the internal circulation of Chinese economy, which must be supplemented by the full use of the international market.

Scholars commonly agree that the primary goal of internal circulation research is to encourage product creation and sales in the domestic market. In this regard, a sound medical insurance scheme will usually cover the treatment of the diseases of the consumers, thus facilitating the social consumption and retail of goods. It is noteworthy that foreign scholars first studied the impact of demand on economic development. Following the same trend, researchers such as Chenery and Syrquin (8) focused on the contribution of consumer demand toward economic growth and believed that economic growth could not be separated from the expansion of demand and structural transformation. While Colm et al. (9) believed that demand can influence economic growth by influencing the supply. Furthermore, in their study, Chenery (10) and Garegnani (11) demonstrated that demand scale and structural changes are the main characteristics of economic development in developing countries. According to the prospect theory proposed by Kahneman and Tversky (12), consumption is likely to produce a positive ratchet effect by improving supply and demand and their mutual promotion. In another instance, Hesketh et al. (13) also noted that a sound system of health insurance can affect the economic development level and consumption, given that further research is conducted in the area. In addition to this, Blinder-Oaxaca also used the classical wage differential decomposition method to demonstrate the impact of health insurance on the economy. In another instance, researchers such as Fairlie (14) and Bauer and Sinning (15) further, extended this method to the non-linear regression model to demonstrate the impact of health insurance on the economy. Moreover, research conducted by Dizioli and Pinheiro (16) showed that the rural medical insurance system can significantly increase the agricultural labor hours of rural residents and, at the same time, improve the labor supply and supply efficiency of the rural residents (17).

According to the research of Forder (18), medical insurance can significantly reduce the economic burden of patients and relieve the economic pressure as well. Also, Hughes et al. (19), through the study of Chicago Medical Insurance Medium- and Long-Term Home Care Program, demonstrated a significant reduction in family expenses. While Forder (18) tested the substitution effect of long-term care on the hospitalization behavior of the elderly in the UK and found that, for every extra pound of nursing, hospital expenses decreased by 0.35 pounds. Goda (20) also found that the insurance security system can reduce family expenses. In another instance, Kim and Lim (21) studied the medical insurance system in South Korea and found that medical insurance subsidies significantly reduced the cost of treatment for individuals with severe disability, which especially entailed the cost of hospitalization. Moreover, Gaughan et al. (17) found that, after the purchase of medical insurance, the number of patients pressing for hospital beds decreased, alleviating the phenomenon of delayed hospitalization. Other than that, Costa-Font et al. (22) deduced data from Spain and found that, after the introduction of public subsidies for long-term care insurance, hospitalization rates and the length of stay had significantly reduced, effectively saving the treatment consumption expenditure.

If looked in depth, scholars from a different angle of the qualitative analysis that is based on the economic growth of medical treatment insurance, especially the domestic product purchase and consumption of in-depth studies, can be found. A few evidence-based medical insurances of the scholars for the promotion of the domestic economic cycle also provide the research angle of view for this article, which makes for a limited level of contribution margin as well.

## Data and Methodology

### Data Selection

To illustrate their shared power relationship, the medical and health care market price index (the same month last year = 100) and the overall retail sales of social consumer products have been used in this study. From the realm of medical insurance research that is mainly concentrated in the social consumer goods purchase, this study selects the monthly data from the time period between January 2010 and January 2021. Moreover, the method of collecting data has been the drawing boots rolling causality, the dynamic test of medical insurance transfer of China in the whole sample, and the subsequent structural changes during the different subsamples that have been taken under consideration. The results are then explained further in the study.

### Research Methods

The testing method that has been resorted to in this study is based on the Granger causality test, and Zhiweisu uses the rolling window mode to test the causality of the samples. The time series tested by the Granger causality test hypothesis is also stable in nature. However, when and if the premise of this hypothesis is not established, the statistics of the full-sample causality test will no longer obey the standard asymptotic distribution, and the estimation of the Vector autoregression (VAR) model will also cause potential difficulties. In this study, the relationship between the medical insurance expenditure and the internal circulation of goods has been tested using the Residual-Based Bootstrap corrected LR statistics method. The retail price index for the medical and wellness products is represented by MED, and the overall retail sales of social consumer goods, through consumer goods in domestic trade are represented by CON.

The following equation shows the generation of the VAR (p) model, which will be used in this study to explain the causality test of the LR statistics, based on the RB correction.

(1) $$\begin{array}{l} Y_{t}=\phi_{0}+\phi_{1}y_{t-1}+\ldots \phi_{p}y_{t-p}+\varepsilon_{t},t=1,2,\ldots ,T \end{array}$$

In this study, the white noise matrix is denoted by εt = (ε1t, ε2t)′, with a zero mean and zero covariance. Moreover, the subscript P represents the optimal lag period, as determined by the Schwartz Information Criteria (SIC). Also, with Variable Yt = (medt, cont), Equation (1) is expressed as:

(2) $$\begin{array}{l} \left[\begin{array}{c} med_{t}\\ con_{t} \end{array}\right]=\left[\begin{array}{c} \varphi_{10}\\ \varphi_{20} \end{array}\right]+\left[\begin{array}{cc} \varphi_{11}\left(L\right) & \varphi_{12}\left(L\right)\\ \varphi_{21}\left(L\right) & \varphi_{22}\left(L\right) \end{array}\right]\left[\begin{array}{c} med_{t}\\ con_{t} \end{array}\right]+\left[\begin{array}{c} \varepsilon_{1t}\\ \varepsilon_{2t} \end{array}\right] \end{array}$$

Moving on, Medt represents the medical insurance expenditure, while the consumption expenditure is given by con_t_, φ_ij_(L) = $\sum_{k=1}^{p+1}\varphi_{ij}$, _k_L^k^, i, j = 1, 2 hysteresis operator is abbreviated by L, $L^{k}X_{t}=X_{t-k}$.

In this study, we let φ_12_, _k_ = 0 (k = 1, 2, 3, …, *p*), to be the null hypothesis that proposes that consumption is not the Granger cause of medical expenditure and can be tested. Moving on, we let φ_21_, _k_ = 0 (k = 1, 2, 3, …, *p*), Granger causality as the null hypothesis that proposes that medical expenditure is not a part of consumption, and thus, this can be tested. Furthermore, the full sample causality has been tested by the probability value p of RB and the modified LR statistics. The null hypothesis, φ_12_, _k_ = 0 (k = 1, 2, 3, …*, p*) has thus been rejected, proving that consumption has a significant causal relationship with medical care. This primarily indicates that consumers can effectively explain changes in medical expenditure. Moreover, if φ_21_, _k_ = 0 (k = 1, 2, 3, …, *p*), is rejected, it proves that medical treatment has a significant causal relationship with consumption. This essentially indicates that medical treatment can effectively explain consumption changes.

In the time series causality test, the full-sample causality tends to show changes at the time cutoff point. For this reason, the Sup-F, Exp-F, and Mean-F variables have been used in this study to test the parameter stability of the model. According to the LR statistical sequence, the stability of the parameter tends to change abruptly at unknown time nodes. For this purpose, the Monte Carlo simulation has been carried out on the whole sample of the parameter VAR model to calculate the mean value and to form an asymptotic distribution.

For virtual variables and the sample separation prior to the deviation, the structural change inspection technology in this study, which is based on the revised dial boots, is estimated using the sample points causality test rolling window. This would have divided the whole, complete samples into smaller causality test samples. While the small sample from the whole samples in the time series (T), set samples of length L, each subsample end π = L, and L +1... T, which would construct the T-1 subsamples. By modifying the LR causality test with RB, the empirical results of the causality test can therefore be obtained in all the considered samples. Thus, the following equation describes the effect of consumption on the price of health care:

(3) $$\begin{array}{l} I\left(1\right)=N_{\text{b}}^{-1}\overset{p}{\underset{k-1}{\sum }}\varphi_{12,\;k}^{\times } \end{array}$$

where N_b_ denotes the number of plucking repeats, and $\varphi_{12,\;k}^{\times }$ is the estimation of plucking in the VAR model. Equations (2, 3) thus, analyze the impact of medical treatment on consumption where the function $\varphi_{12,\;k}^{\times }$ is the estimation of boots in the VAR model.

(4) $$\begin{array}{l} I\left(2\right)=N_{\text{b}}^{-1}\overset{p}{\underset{k-1}{\sum }}\varphi_{21,\;k}^{\times } \end{array}$$

According to the estimation of the boot repetition, each incremental interval in the regression tends to have an impact on the accuracy of the empirical results. Therefore, smaller increment intervals have the capability of improving the inspection accuracy of the rolling window and simultaneously ensuring the exact number of observations of the VAR model.

## Results

### Full Sample Causality Test

In this study, the unit root test was carried out first. In this regard, results of the NG test and the ADF test showed that the null hypothesis was rejected at a 99% confidence level, and both of them were zero-order single integral sequences. Therefore, keeping this in check, the Granger causality test for the full sample of the boot pulling could thus be carried out, with details shown in Table 1.

**Table 1:** Granger causality test for the whole sample of boots.

**Null hypothesis**	**Health care does not affect consumption**		**Consumption does not affect health care**	
	**Statistic**	***P*-value**	**Statistic**	***P*-value**
Test result	25.0741	0.0000	0.3947	0.55

As shown in Table 1, the consumption does not affect the medical statistics, and the *p*-value results accept the null hypothesis that proposes that consumption does not have a causal relationship with medical treatment. Thus, the results do not appear to have Granger causality. The traditional testing method assumes that there is no structural change observed and that the causal relationship changes due to the time change in the actual sequence. This has been concluded by considering that only a single causal relationship will lead to deviation from reality.

### Stability Test

In this study, three statistics, Exp-F, Mean-F, and Sup-F, were used to test the changes in the consumption and medical expenditure in the abovementioned models. Moreover, the LC was used to test the stability of the parameters in the VAR. The results for this have been displayed in Table 2.

**Table 2:** Stability test.

	**Medical insurance expenditure equation parameter**		**Consumption expenditure equation parameter**		**VAR system parameter**	
	**The statistical value**	***P*-value**	**The statistical value**	***P*-value**	**The statistical value**	***P*-value**
Sup-F	6.771	0.001	71.652	0.004	21.118	0.000
Mxp-F	4.323	0.000	5.741	0.021	16.652	0.000
Exp-F	3.784	0.003	42.489	0.038	19.852	0.000
LC test	2.562	0.005	0.560	0.102	4.564	0.000

As shown in the table, according to the parameters of the stability test, it was shown that, under a 90% confidence level to accept the null hypothesis, the results of the inspection of structural change of the whole sample analysis cannot describe the medical expenditure and consumer spending in the complete time series precision Granger causality of the whole sample. Moreover, the VAR model to estimate parameters in the table is not stable, which is necessary for the sample points and Granger causality test.

### Sample Causality Test of Boot Rolling

**Figure 1:**
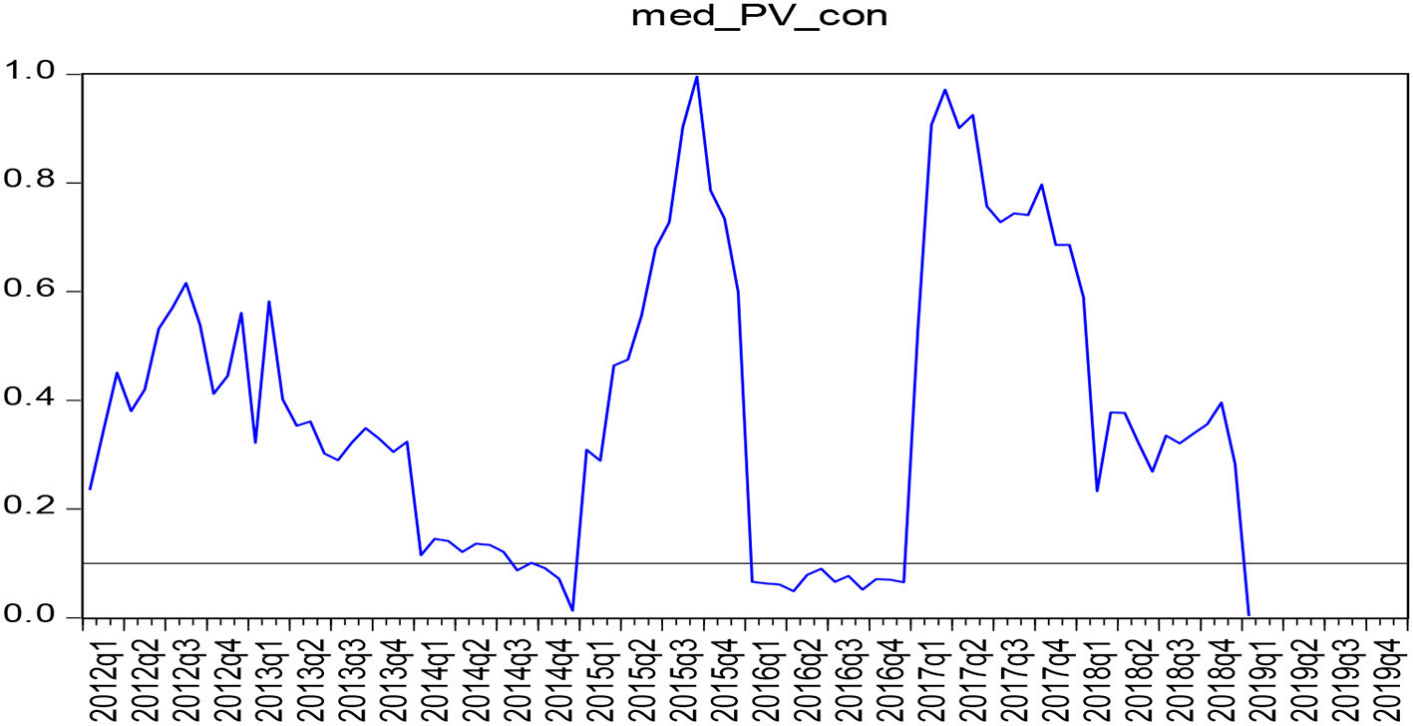
*P*-value diagram of the sample test for the causality of boot-rolling (medical expenditure - consumption expenditure).

**Figure 2:**
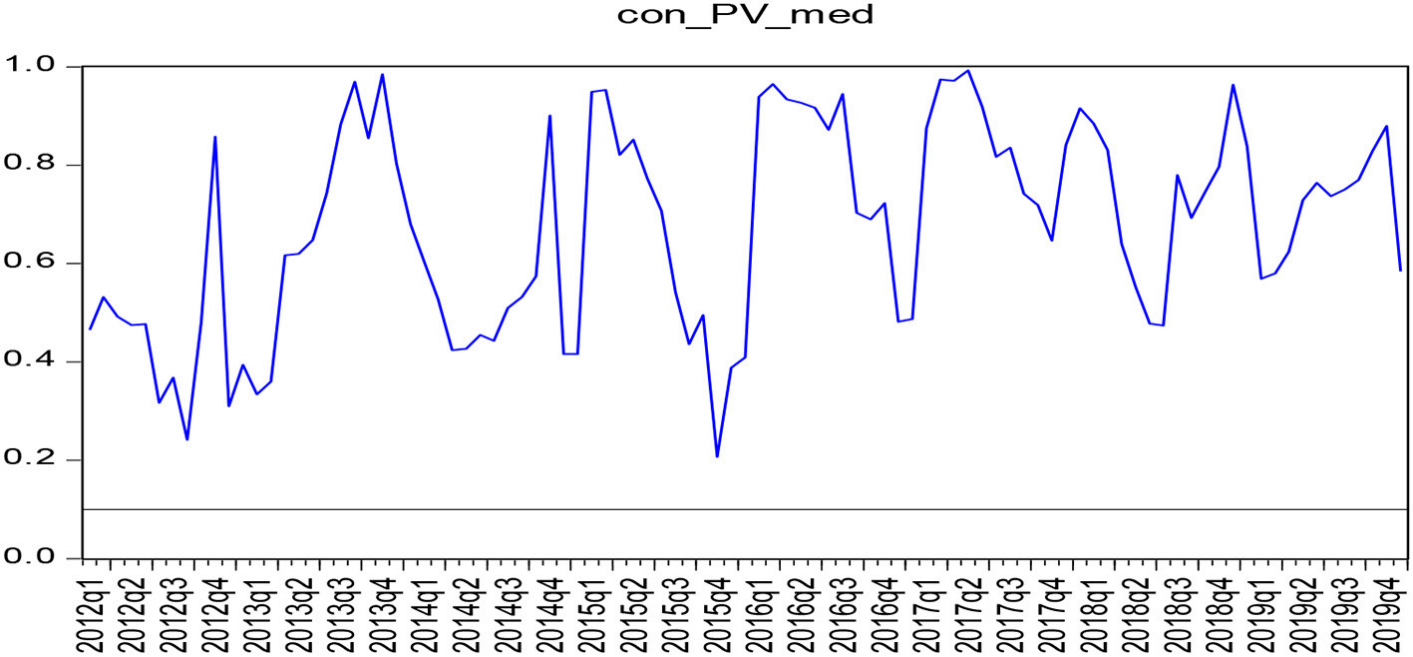
*P*-value diagram of the causality test of shoe rolling sample (consumer expenditure - medical expenditure).

According to the parameters stability test results, medical care and consumption tend to influence the structural changes in the actual situation, and the Granger causality between them cannot be described when the full sample is selected from January 2010 to January 2020. To accurately obtain the causal relationship between the two, this study resorted to the use of the boot-pulling rolling causal relationship to test the causal relationship between January 2010 and January 2020, as shown in Figures 1, 2.

As shown in Figures 1, 2, the test results show that the range of *P*-value <0.1 indicates the rejection of the null hypothesis that proposes that there is no single causal relationship between the two, indicating that medical expenditure thus has a causal relationship with consumption. Moreover, there was a Granger causality relationship between the medical expenditure and consumption expenditure from July 2014 to January 2015, January 2016 to December 2016, and January 2019 to December 2019. There is, however, no Granger causality observed between the consumer expenditure and medical expenditure from January 2010 to December 2019. Also, there is no Granger causality between the consumption expenditure and the medical expenditure in both the full sample and the boot-rolling subsample that have been taken into consideration.

**Figure 3:**
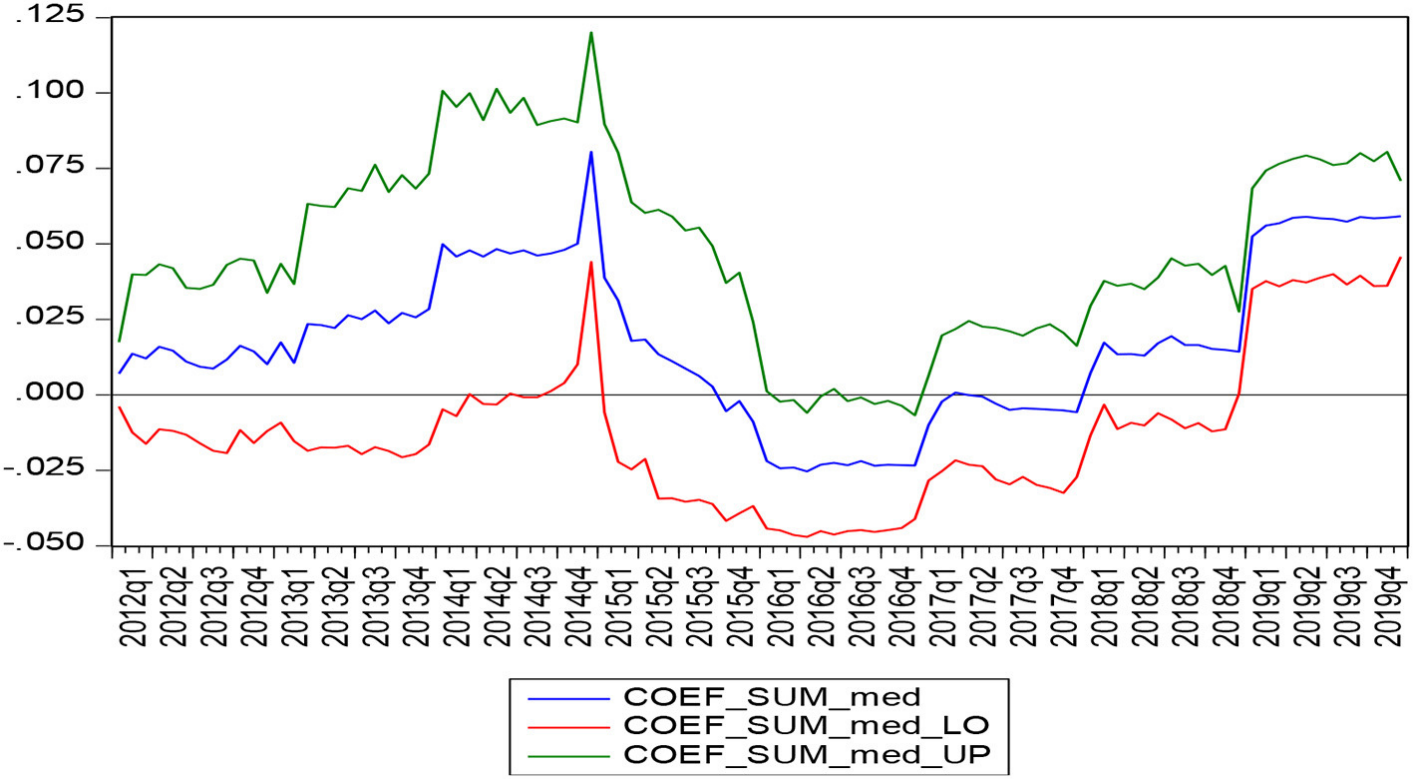
Estimated results of boot rolling (medical expenditure - consumer expenditure).

To test the positive and negative influences of medical expenditure on consumption expenditure, this study thus continues to put the boot-pulling rolling estimation to test. The result of this test had been displayed in Figure 3. It can be observed that the zero lines have been taken as the critical line. When the coefficient is above the zero lines, it indicates that medical expenditure positively impacts the consumption expenditure. Moreover, when the coefficient is below the zero lines, it indicates that the medical expenditure has a negative impact on consumption expenditure. As observed, in the case of this particular study, the coefficient was below the threshold from October 2015 to January 2018, indicating that medical expenditure thus had a negative impact on the consumption expenditure, while the rest of the time period that was taken into consideration left a positive impact.

## Discussion

### Empirical Analysis

The abovementioned experimental results show that the Granger causality between medical expenditure and consumption expenditure does exist. In 2012, China held the 18th National Congress of the Communist Party of China. This was undertaken unswervingly along the path of socialism with Chinese characteristics, to build a well-off society in an all-round way, to put forward the integration of basic endowment insurance and the medical insurance system of the urban and rural residents. This initiative was undertaken to promote medical security, to perfect the system of universal health care, and to establish a guaranteed mechanism toward curing and managing acute diseases. From the perspective of the variable pertaining to the income range, this policy significantly improves the utilization level of the medical services as well. This study has speculated that the primary reason for this result could potentially be that the implementation of medical insurance for urban and rural residents in China has brought about significant changes in the actual compensation rate, especially for the urban residents who have benefited significantly. However, urbanization of China was still not high at that time, and the urban-rural duality was considered to be a serious issue, which hindered the benefits obtained from medical insurance. This was likely to be the policy implication from the Obama government. However, since the time that Donald Trump took the office, there was an increase in the tariffs on Chinese trade that was being undertaken with the United States. Moreover, there was also a hi-tech blockade policy to curb the economic development of China, due to which the Chinese economy suffered serious impact in terms of their export trade, the collapse of the export processing trade type enterprise, and the negative effects on the enterprise workers. Other than that, part of the unemployed workers also had no access to health care, which largely prevented consumer spending in China.

To this end, the Chinese government is continuing to promote the “area” strategy, which was launched in 2016, and focuses on the integration of basic medical insurance systems of the urban and rural residents. The Chinese government has been putting forward the integration of basic medical insurance of urban residents and the new rural cooperative medical care system. They have mainly done this by establishing the “Rural Revitalization Strategic Plan (2018–2022)” and the “Establishment of a Central Adjustment System for Enterprise Employees' Basic Pension Insurance Fund” strategies, which were introduced in 2018. These effective policies and measures have significantly promoted the development of medical undertakings, enabling people to resume the act of consumption, especially after having medical security, and have greatly promoted the consumption expenditure as a result. In May 2020, the Chinese government put forward the strategy of “building a new double-cycle development pattern with internal circulation as the main focus and mutual promotion at home and abroad.” The internal circulation that was undertaken to drive economic growth relies on the internal consumption of China to drive economic development and growth. The empirical results show that a sound medical insurance system can effectively promote consumption expenditure, thus promoting an inner circular economy.

## Conclusion and Policy Suggestions

In the year 2020, the Chinese government aimed to eradicate poverty and achieve victory in the fight against poverty. At the same time, at the end of 2020, according to the statistical bulletin from the National Bureau of Statistics, China, the permanent population urbanization rate of China was more than 60%, while 40% of the population of China lived in rural areas. In this regard, rural medical insurance and endowment insurance are still a phenomenon that is missing in these areas and thus hinder rural consumer spending, which is imperative to expand the domestic economic cycle. For this to happen, China must first eliminate the differences between urban and rural areas and ensure the allocation of resources as a whole. Moreover, the aim is to develop urban and rural areas in a coordinated manner and implement a relevant strategy for rural revitalization. It must be deeply recognized that medical insurance has a positive distribution effect, “income effect,” and “crowding out effect,” which has the capability of significantly reducing the insured out-of-pocket expenses of people and also increasing the consumption expenditure in other aspects of life.

In addition to this, to restrain the rise of housing prices, there must be an expansion of the consumption capacity of residents, which will lead to an increase in the internal circulation of power. In recent years, the debt of the residential sector of China has expanded rapidly, and the risk of residential debt has also worsened, which is mainly reflected in two aspects. First, the leverage ratio of the residential sector of China is at the highest level among the other emerging market countries. According to the data released by the Bank for International Settlements (BIS), the leverage ratio of the household sector of China (the ratio of household sector debt to GDP) was 55.2% in 2019. In this regard, Dynan and Edelberg (23) (2007–2009) pointed out that the high housing prices in recent years are the main reason why some residents bear heavy mortgage pressure. Exorbitant prices of crowding out from the consumer share and the increasing housing prices increase the housing price to income ratio, weaken the purchasing power of the resident, and hinder the potential demand into actual purchasing power effectively. Moreover, to realize the purchase, increasing housing prices suppress the current consumption and savings, while the real estate spending also increases in order to reduce other consumer goods, which then produces the substitution effect and the crowding effect, and increase the living costs of residents and the consumer spending. Third, there is also a deepening of the supply-side structural reforms as a strategic foundation for expanding the internal cycle.

The integration of supply and demand tends to lay the groundwork for high quality growth in China and then stimulate demand on the demand side. This also expands and upgrades consumption at the same time and builds the demand for domestic industrial chain expansion of China and upgrade. Through independent research and development, China will reduce its dependence on developed countries, promote the upscaling of its domestic industrial chain, and thus create the relevant supply conditions for the upgradation of consumption.

Finally, to increase the good government consumption spending, the internal cycle also needs to support the “two new and one heavy” construction method. The government should ideally increase the effective investment, focusing on supporting the construction of new infrastructure, new urbanization, and major projects related to the national economy and the livelihood of people. Therefore, we should coordinate the efforts to adjust the economic structure, promote industrial transformation and upgradation initiatives, and enhance the core competitiveness of the economy.

### Policy Suggestions

#### New Infrastructure Drives New Consumption

Introduction to new infrastructure will promote and upgrade the consumption levels, improve the consumption demand in China at both the quantity and quality levels, and provide an infrastructure system of digital transformation. It will also aid in innovative upgradation, integrated innovation, and other services and promote industrial upgrading as well. At present, China has yet to make a structural breakthrough in replacing the old drivers of growth with the new ones and is generally at the middle and low end of the global industrial chain. By stimulating the building of new infrastructure and acting as a catalyst for post-epidemic economic transition and social change, the Chinese economy will be able to compensate for inadequacies in the high-end industrial chain and improve its global competitiveness.

#### New Urbanization Drives Economic Growth

In its natural essence, urbanization is also another form of modernization. Urbanization, along with the new infrastructure, is going to be a propelling force in the social and economic development of China. In this context, the maintenance of a basic driving force for the social and economic growth of China is necessary, primarily by promoting high-quality development and population accumulation and improving the economic structure through urbanization. The initiative of new urbanization is likely to promote the integrated development of the city clusters and metropolitan areas. By making the cities more receptive and inclusive of the population, China will be able to achieve better school enrollment, employment, and medical care to promote individual development and overall social development. This new type of urbanization promotes the integration of people and cities, providing a place for new infrastructure to be implemented and creating more value.

#### Major Projects to Improve the Lives of People

Major projects such as transportation and water conservancy are critical to ensure livelihood of people, stabilize expectations, boost consumption, and break the development bottlenecks. In this regard, the use of surplus resources at home and abroad will strengthen major infrastructure construction and improve transportation and water conservancy infrastructure. It will also strengthen the material foundation for new infrastructure construction and new urbanization, boost the development of relevant industries, and create the potential for economic and social development as well. By visibly improving the medical expenditure, the promotion of domestic consumer spending and the promotion of the concept of a recycling economy, domestic consumption will have causality of medical expenditure. Therefore, we should improve the system of medical insurance, make people at ease when it comes to consumption, rein in property prices, expand domestic demand, increase government investment effectively, promote consumption, and finally increase the internal circulation dynamics.

## Data Availability Statement

The original contributions presented in the study are included in the article/supplementary material, further inquiries can be directed to the corresponding author/s.

## Author Contributions

PZ: conceptualization, software, data curation, and writing — original draft preparation. L-YW: methodology, visualization, and investigation. LZ: writing — reviewing and editing. All the authors contributed to the article and approved the submitted version.

## Conflict of Interest

The authors declare that the research was conducted in the absence of any commercial or financial relationships that could be construed as a potential conflict of interest.
